# Regulated necrosis-related molecule mRNA expression in humans and mice and in murine acute tissue injury and systemic autoimmunity leading to progressive organ damage, and progressive fibrosis

**DOI:** 10.1042/BSR20160336

**Published:** 2016-12-09

**Authors:** Mohsen Honarpisheh, Jyaysi Desai, Julian A. Marschner, Marc Weidenbusch, Maciej Lech, Volker Vielhauer, Hans-Joachim Anders, Shrikant R. Mulay

**Affiliations:** * Medizinische Klinik und Poliklinik IV, Klinikum der Universität München, Ludwig Maximilians University of Munich, Munich, 80336 Germany

**Keywords:** fibrogenesis, inflammation, kidney injury, necroinflammation, necroptosis, regulated cell death

## Abstract

The species-specific, as well as organ-specific expression of regulated necrosis (RN)-related molecules, is not known. We determined the expression levels of tumour necrosis factor receptor-1 (TNFR1), receptor activated protein kinase (RIPK)1, RIPK3, mixed lineage kinase domain-like (MLKL), CASP8, Fas-associated protein with death domain (FADD), cellular inhibitor of apoptosis protein (CIAP)1, CIAP2, glutathione peroxidase-4 (GPX4), cyclophilin D (CYPD), CASP1, NLRP3 and poly(ADP-ribose) polymerase-1 (PARP1) in human and mouse solid organs. We observed significant differences in expression of these molecules between human and mice. In addition, we characterized their expression profiles in acute as well as persistent tissue injury and chronic tissue remodelling using acute and chronic kidney injury models. We observed that the degree and pattern of induction of RN-related molecules were highly dependent on the trigger and disease pathogenesis. Furthermore, we studied their expression patterns in mice with lupus-like systemic autoimmunity, which revealed that the expression of MLKL, GPX4 and PARP1 significantly increased in the spleen along disease progression and CASP1, RIPK1, RIPK3 and CYPD were higher at the earlier stages but were significantly decreased in the later stages. In contrast, in the kidney, the expression of genes involved in pyroptosis, e.g. NLRP3 and CASP1 were significantly increased and TNFR1, RIPK1, RIPK3, CIAP1/2 and GPX4 were significantly decreased along the progression of lupus nephritis (LN). Thus, the organ- and species-specific expression of RN-related molecules should be considered during designing experiments, interpreting the results as well as extrapolating the conclusions from one species or organ to another species or organ respectively.

## INTRODUCTION

For almost two decades apoptosis was considered to be the only programmed form of cell death and necrosis was felt to be an ‘accidental’ cell death passively induced by physiochemical insults [1]. However, recent evidence revealed multiple pathways of regulated necrosis (RN) that share common morphological features e.g. cellular leakage, cytoplasmic granulation and organelle and/or cellular swelling [2]. Based on the engagement of distinct signalling pathways, RN is currently categorized as necroptosis, ferroptosis, mitochondria permeability transition (MPT)-RN, pyroptosis, parthanatos, catastrophic mitosis, podoptosis and neutrophil extracellular trap (NET)-associated cell death, NETosis etc. [2,3].

### Necroptosis

Necroptosis is triggered by death receptors and requires the receptor activated protein kinase (RIPK)3-dependent phosphorylation of mixed lineage kinase domain-like (MLKL) inducing plasma membrane pore formation [4,5]. Tumour necrosis factor receptor-1 (TNFR1)-mediated necroptosis is considered a prototype form of RN. TNFR1 stimulation recruits RIPK1, which possesses important kinase-dependent and scaffolding functions that either inhibit or trigger necroptosis and apoptosis [6]. Upon deubiquitination, RIPK1 is translocated to the cytosol where it targets RIPK3 via the RHIM domain [2]. TNFR1 activation may also lead to apoptosis; however, the deficiency of caspase-8 [7], a Fas-associated protein with death domain (FADD) [8], FLICE-like inhibitory protein [9] and cellular inhibitor of apoptosis proteins (CIAPs) 1 and 2 [10] induce necroptosis. In addition to the death receptors, interferons, toll-like receptors (TLRs), intracellular RNA or DNA sensors as well as inorganic crystals trigger necroptosis [11,12]. Furthermore, distinct stimuli involve the necroptosis pathway into NET formation-related neutrophil death, known as ‘suicidal NETosis’ [13,14].

### Ferroptosis

Iron-dependent accumulations of intracellular lipid reactive oxygen species (ROS) lead to cellular necrosis, which is termed as ferroptosis [15]. Ferroptosis is triggered by chemical inhibition of the cellular cystine/glutamate antiporter system X_c,_ using erastin, leading to depletion of intracellular generation of GSH, an antioxidant. Glutathione peroxidase-4 (GPX4) reduces hydrogen peroxide to water using GSH and therefore, suppression of GPX4 results in ferroptosis [16].

### MPT-RN

Under stressful cellular conditions, MPT is accompanied by the unspecific opening of the MPT pore on the inner membrane of mitochondria leading to necrosis, termed as MPT-RN [2]. Cyclophilin D (CYPD) is an important regulator of the pore opening since both genetic deficiency and chemical inhibition of CYPD have been shown to protect cells from MPT-RN [17–19].

### Pyroptosis

Pyroptosis is a proinflammatory form of RN. The activation of NLRP3 or other inflammasomes leads to caspase-1 activation that cleaves the pro-IL1β and proIL-18 into their active subunits to allow their cellular release by cell membrane rupture, termed as pyroptosis [2,20,21].

### Parthanatos

Poly(ADP-ribose) polymerase-1 (PARP1) overactivation causes PARylation of proteins that deplete cells of NAD^+^ and induce RN, termed as parthanatos [2,22].

RN and inflammation can induce each other in an auto-amplification loop of necroinflammation resulting in exaggerated cell death and inflammation that may lead to organ failure [23–25]. Since, most experiments are conducted in mice the relevance for the human disease remains a concern, and discrepancies in organ-specific expression levels between species were previously shown for pattern recognition receptors (PRRs), C-type lectins and TLR accessory molecules [26–31]. We hypothesized the same for the RN-related molecules, and hence, determined their mRNA expression profiles in human and mice organs as well as in murine autoimmunity, acute tissue injury and progressive tissue fibrosis.

## MATERIALS AND METHODS

### Human solid organ total RNA and cDNA preparation

All human solid organ total RNA from pools of healthy human tissues were purchased from Clontech. An equal amount of total RNA from each individual sample was used as a template in cDNA preparation with Superscript II (Invitrogen). As only a single pool was available for each organ, no studies on biological replicates allowing statistics could be performed. According to Clontech, all human organ samples were purchased and imported in accordance with all local laws and regulations.

### Mouse solid organ cDNA preparation for qRT-PCR experiments

Ten to twelve-week old adult C57BL/6 male mice were obtained from Charles River. Mice were housed in groups of five under specific pathogen-free conditions with unlimited access to food and water. Mice were killed by cervical dislocation. RNA was isolated from freshly harvested tissues as described [30]. All organs were kept in RNA later solution and RNA was isolated with an equal amount of tissue mass. RNA was purified with Pure Link RNA Mini Kit (Ambion) according to manufacturer's instructions. All RNA samples were digested with DNAse enzyme and additional washing steps were performed to remove traces of DNAse. RNA concentrations were measured with NanoDrop 1000 Spectrophotometer. Only samples with absorbance 260/280 between 1.95 and 2.05 were considered as pure RNA, the integrity of the total RNA was determined by electrophoresis on 2% (w/v) agarose gels as described.

### Animal studies

Groups of 7–8-week old C57BL/6 male mice (*n*=5–10) underwent bilateral or unilateral renal pedicle clamping for 35 min followed by reperfusion as a model of ischaemia-reperfusion as described [32,33]. Body temperature was maintained at 37°C throughout the procedure using a heating plate (Kleintier-OP-Tisch M12511, Medax) and an egg breeding device (Octagon 20 Advance, Brinsea Products). Mice underwent bilateral pedicle clamping were killed after 24 h of reperfusion to study acute ischaemic injury whereas mice underwent unilateral pedicle clamping were killed at day 5, 10 and 35 to study CKD after AKI. Injured and contralateral kidneys were harvested for RNA isolation and histology analysis. Contralateral kidneys served as internal control kidneys. To induce acute oxalate nephropathy, mice received a single intraperitoneal injection of 100 mg kg^−1^ of sodium oxalate (Santa Cruz Biotechnology) and 3% sodium oxalate in drinking water and kidneys were harvested after 24 h for RNA isolation and histology analysis [11,34]. For cisplatin nephropathy model, mice received a single intraperitoneal injection of 20 mg kg^−1^ of cisplatin (Sigma–Aldrich) and kidneys were harvested after 3 days for RNA isolation and histology analysis [35]. Chronic oxalate nephropathy was induced by feeding mice an oxalate-rich diet that was prepared by adding 50 μmol/g sodium oxalate to a calcium-free standard diet (Ssniff) as recently described [36]. Mice were killed at day 7, 14 and 21 [36]. For the lupus model, groups of female MRL/WT or MRL/lpr mice with spontaneous lupus-like autoimmunity were killed at 6, 10 and 14 weeks as described [37,38]. MRL/lpr female mice develop a lupus-like disease at week 10, which is further aggravated in the next weeks. Thus, week 14, a time point when the mice already show fully developed the lupus-like disease, was chosen. Week 6 was used as a baseline control [38]. Kidneys and spleens were harvested for RNA isolation and RT-PCR. All experimental procedures were performed according to the German animal care and ethics legislation and had been approved by the local government authorities.

### Quantitative real time-PCR

An equal amount of RNA (1 μg) was used to prepare cDNA [31]. Complementary DNA was performed with Superscript II reverse transcriptase (Thermo Fisher), 5× first-strand buffer (Thermo Fisher), DTT (Invitrogen), dNTPs (GE Healthcare), linear acrylamide (Ambion), hexanucleotide (Roche) and RNasin (Promega). Reverse transcriptase reaction was performed for 90 min at 42°C then the reaction was heated at 85°C for 5 min using a Mastercycler pro (Eppendorf). RN-related molecule mRNA expression in human and mouse solid organs, as well as diseases model, were quantified by RT-PCR using GAPDH as housekeeper gene for human samples and 18s rRNA for mouse samples as described previously [30]. Each PCR reaction (20 μl) involved 10× *Taq* Polymerase Buffer, *Taq* Polymerase, dNTPs, BSA, PCR Optimizer, SYBR Green dye, MgCl_2_, gene specific primers and 0.2 μl of synthesized cDNA. SYBR Green dye fast two-step detection protocol from Light Cycler 480 (Roche) running programme was used for amplification. Each amplification step included initiation step 95°C, annealing step 60°C and amplification step 72°C and was repeated 40 times. Gene-specific primers (300 nM, Metabion) were used as listed in Table 1. Double-distilled H_2_O was used as negative control for target and housekeeper genes. Specific primer for each target was designed using the primer designing tool (NCBI) and *in silico* specificity screen (BLAST) was performed. The lengths of amplicons were between 90 and 120 bp. The kinetics of the PCR amplification (primer efficiency) was calculated for each set of primers. The efficiency-corrected quantification was performed automatically by the Light Cycler 480 based on extern standard curves describing the PCR efficiencies of the target and the reference gene [ratio=E_target_^ΔCPtarget (control − sample)^/E_ref_^ΔCPref (control − sample)^]. The high confidence algorithm was used to reduce the risk of the false positive crossing point. All the samples that rise above the background fluorescence (crossing point *C*_p_ or quantification cycle *C*_q_) between 5 and 40 cycles during the amplification reaction were considered as detectable. The melting curves were analysed for every sample to detect unspecific products and primer dimers. To visualize the similarity and differences in gene expression profiles among the samples, hierarchical cluster analysis was performed using algorithms incorporated in the open-source software MultiExperiment Viewer (MeV) version 4.9 [39]. Differentially expressed mRNAs were screened by Volcano Plot between log 2 (fold change) gene expression [unstandardized signal] against −log 10 (*P*-value) from the *t* test [noise-adjusted/standardized signal] [40].

**Table 1:** Primers used for RT-PCR

Heading	Forward sequence (5′→3′)	Reverse sequence (3′→5′)	Accession number	Efficiency
**Human**				
TNFR1	CCGCTTCAGAAAACCACCTCAG	ATGCCGGTACTGGTTCTTCCTG	NM_001065	2.00
RIPK1	TATCCCAGTGCCTGAGACCAAC	GTAGGCTCCAATCTGAATGCCAG	NM_003804	2.24
RIPK3	GCTACGATGTGGCGGTCAAGAT	TTGGTCCCAGTTCACCTTCTCG	NM_006871	2.34
MLKL	TCACACTTGGCAAGCGCATGGT	GTAGCCTTGAGTTACCAGGAAGT	NM_152649	1.98
CASP8	AGAAGAGGGTCATCCTGGGAGA	TCAGGACTTCCTTCAAGGCTGC	NM_033355	2.06
FADD	GCTTCTGAACTCAAGCTGCG	CCCGCAGGAGCTCATTTAGT	NM_003824	1.97
CIAP1	CAGACACATGCAGCTCGAATGAG	CACCTCAAGCCACCATCACAAC	NM_001166	1.88
CIAP2	GCTTTTGCTGTGATGGTGGACTC	CTTGACGGATGAACTCCTGTCC	NM_001165	2.05
GPX4	ACAAGAACGGCTGCGTGGTGAA	GCCACACACTTGTGGAGCTAGA	NM_002085	1.88
CYPD	CAGAATGGGACAGGTGGAGAAAG	CTGAGAACCGTTTGTGTTGCGG	NM_005038	2.07
CASP1	GCTGAGGTTGACATCACAGGCA	TGCTGTCAGAGGTCTTGTGCTC	NM_001257118	1.88
NLRP3	GGACTGAAGCACCTGTTGTGCA	TCCTGAGTCTCCCAAGGCATTC	NM_001079821	2.09
PARP1	CCAAGCCAGTTCAGGACCTCAT	GGATCTGCCTTTTGCTCAGCTTC	NM_001618	2.10
**Mouse**				
TNFR1	GTGCGTCCCTTGCAGCCACT	GCAACAGCACCGCAGTAGCTGA	NM_011609.4	2.20
RIPK1	GACTGTGTACCCTTACCTCCGA	CACTGCGATCATTCTCGTCCTG	NM_009068	1.94
RIPK3	GAAGACACGGCACTCCTTGGTA	CTTGAGGCAGTAGTTCTTGGTGG	NM_019955	2.22
MLKL	CTGAGGGAACTGCTGGATAGAG	CGAGGAAACTGGAGCTGCTGAT	NM_001310613	2.25
CASP8	ATGGCTACGGTGAAGAACTGC	TAGTTCACGCCAGTCAGG	NM_009812	2.20
FADD	CACACAATGTCAAATGCCACCTG	TGCGCCGACACGATCTACTGC	NM_01017	2.02
CIAP1/2	GGACATTAGGAGTCTTCCCACAG	GAACACGATGGATACCTCTCGG	NM_007464	2.20
GPX4	CCTCTGCTGCAAGAGCCTCCC	CTTATCCAGGCAGACCATGTGC	NM_001037741	2.17
CYPD	GGACGCTTGAAAATGTAGAGGTG	GGATGACTGTCACCAGAGCCAT	NM_026352	2.27
CASP1	TCAGCTCCATCAGCTGAAAC	TGGAAATGTGCCATCTTCTTT	NM_009807	2.01
NLRP3	TCACAACTCGCCCAAGGAGGAA	AAGAGACCACGGCAGAAGCTAG	NM_145827	2.04
PARP1	CTCTCCCAGAACAAGGACGAAG	CCGCTTTCACTTCCTCCATCTTC	NM_007415	2.15

### Histopathology

The harvested kidney tissues were fixed in 4% neutral-buffered formalin followed by dehydration in graded alcohols and embedded in paraffin. For periodic acid–Schiff (PAS) and haematoxylin and eosin (H&E) staining or immunostaining, 4 μm sections were deparaffinized, rehydrated, transferred into citrate buffer and either autoclaved or microwave treated for antigen retrieval and processed as described [12]. Tubular injury was scored by assessing the percentage of necrotic tubules and presence of tubular casts. For immunostaining, following primary antibodies were used: RIPK3, MLKL and SMA (Abcam), Collagen1α1 (Dako).

### Statistics

Data were expressed as mean ± S.E.M. Comparisons between groups were performed using unpaired Student's two-sided *t* test. A *P*-value less than 0.05 indicated statistical significance.

## RESULTS

### Regulated necrosis-related molecule mRNA expression in adult human tissues

**Figure 1:**
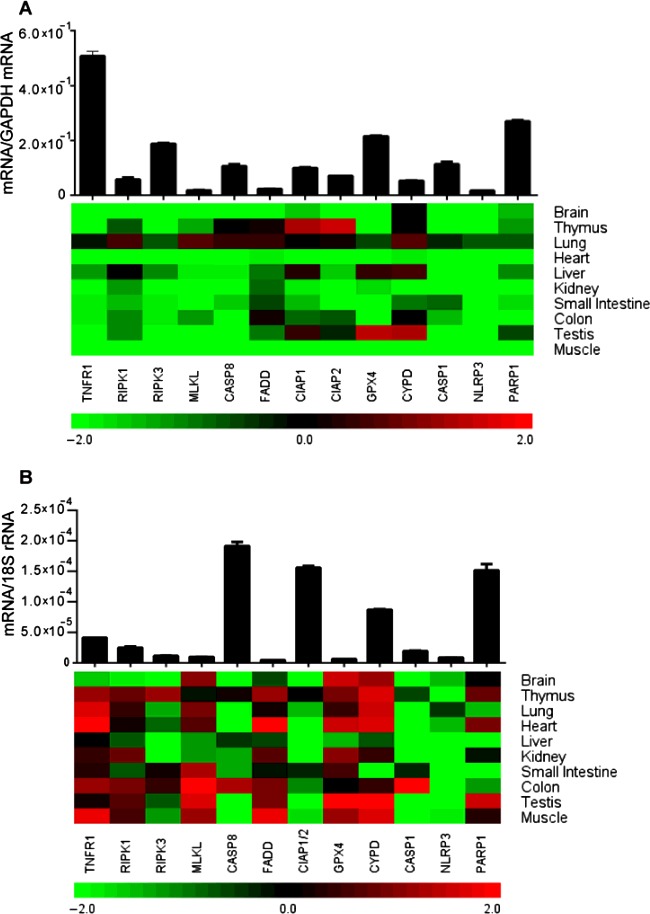
RN-related molecules mRNA expression in adult human and mouse tissue (**A**) Quantitative real-time PCR analysis was performed on cDNAs prepared from pools of healthy human tissue as described in the ‘Materials and Methods’ section and mRNA expression levels of all the organs were normalized to GAPDH mRNA expression level and spleen mRNA levels were illustrated in the form histograms. Whereas the mRNA expression levels of other organs were normalized to spleen mRNA expression levels and were represented in the heatmap. (**B**) cDNAs derived from five adult 12-week old C57BL/6 mice as described in ‘Materials and Methods’ section and mRNA expression levels of all the organs were normalized to 18s rRNA expression level and the spleen mRNA levels were illustrated in the form of histograms. Whereas the mRNA expression levels of respected other organs were normalized to spleen expression levels and were represented in the heatmap. Data represent means ± S.E.M.

We quantified the mRNA expression levels of RN-related molecules involved in necroptosis, e.g. TNFR1, RIPK1, RIPK3, MLKL, CASP8, FADD and CIAP1 and CIAP2; ferroptosis, e.g. GPX4; MPT-RN, e.g. CYPD; pyroptosis, e.g. CASP1, NLRP3; parthanatos, e.g. PARP1 using qRT-PCR in healthy human organs. All of these molecules were constitutively expressed in the human spleen; however, the mRNA expression levels of MLKL, NLRP3 and FADD were generally low (Figure 1A). GPX4 and CYPD expressions were higher in testis, whereas expression of CIAP1 and CIAP2 was higher in the thymus but all remained low in any other solid organs tested. Lung expressed higher levels of RIPK1, MLKL and CYPD. The later was also expressed at higher levels in the liver. Further, higher expression of CIAP1 was observed in testis. Apart from this, the mRNA expression of most of the other molecules was markedly lower in the solid organs compared with spleen. Thus, mRNA expression levels of most RN-related molecules are low in healthy solid organs compared with spleen except for CYPD in testis and CIAP1/2 in the lung.

### Regulated necrosis-related molecule mRNA expression in adult murine tissues

**Figure 2:**
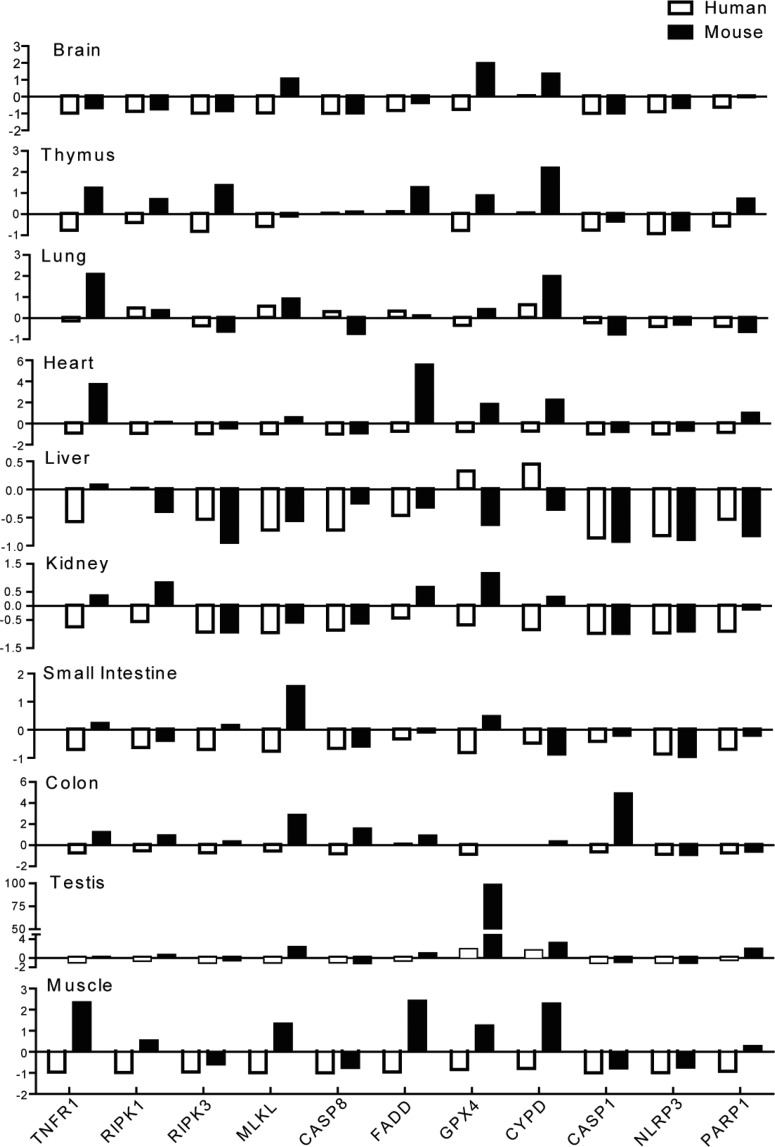
The relative expression of RN-related molecules mRNA in different organs compared with the spleen The respective relative human (white bars) and murine (black bars) and RN-related molecular mRNA levels from Figure 1 are illustrated. The *x*-axis shows mRNA expression levels of the organs normalized to spleen mRNA expression levels of the respective species. The *y*-axis marks the fold-change in each direction. Note that the scale of the *y*-axis is different for each organ. Data represent means ± S.E.M.

Next, we examined the same organs from healthy, 10–12 weeks old, C57BL/6 mice to quantify the mRNA expression levels of the RN-related molecules as listed earlier. All molecules were constitutively expressed in mouse spleen; however, the mRNA expression levels of RIPK3, MLKL, FADD, GPX4 and NLRP3 were low (Figure 1B). Unlike human solid organs, RN-related molecule mRNA expression varied between murine solid organs compared with the spleen. NLRP3, RIPK3, CASP1, CASP8 and CIAP1/2 mRNA expression levels were very low in all the solid organs tested as compared with spleen except for RIPK3 in thymus, CASP1 and CASP8 in the colon and CIAP1/2 in thymus, heart and testis. MLKL mRNA was expressed at higher levels in the brain, small intestine, colon, testis and muscle. TNFR1 mRNA levels were higher in thymus, lung, heart, colon and muscles, whereas GPX4 mRNA levels were higher in the brain, thymus, heart, kidney, testis and muscle. Brain, thymus, lung, heart, testis and muscles expressed higher mRNA levels of CYPD. Thymus, heart and muscles expressed high levels FADD mRNA. RIPK1 mRNA was only moderately expressed in thymus, heart, kidney, colon and testis. PARP1 was highly expressed only in the testis and was expressed at moderately high levels in thymus and heart. Figure 2 compares the organ-specific RN-related molecule mRNA expression in humans (white bars) and mice (black bars). The graphs illustrate the obvious differences in relative mRNA expression between humans and mice. For example, TNFR1, MLKL and CYPD mRNA expressions were much higher in murine organs. Human lung, liver and testis express higher relative levels of CYPD mRNA compared with other human solid organs. All organs except colon and liver expressed high levels of GPX4 mRNA. Interestingly, testis express remarkably high (up to 97-fold) levels of GPX4 mRNA compared with the spleen. Thus, the relative mRNA expression levels of RN-related molecules largely differ between healthy human and mouse organs.

### Regulated necrosis-related molecule expression in acute tissue injury in mice

**Figure 3:**
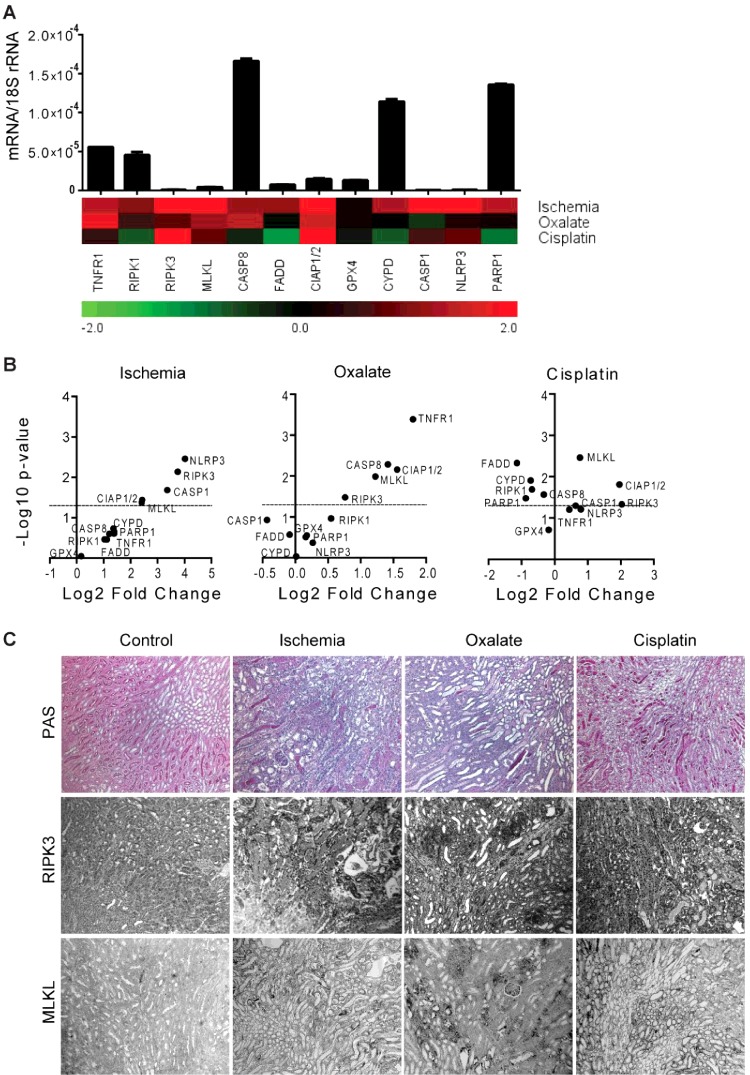
RN-related molecules mRNA expression in AKI Animal models of AKI were induced as described in ‘Materials and Methods’ section. (**A**) Quantitative RT-PCR was performed on cDNAs derived from the injured kidneys. The mRNA expression levels were normalized to 18s rRNA expression level. The histogram represents the mRNA expression levels of different genes of the wild-type kidney (control). Data represent means ± S.E.M. The heatmap represents the relative expression of mRNA levels in different models compared with control. (**B**) The statistical analysis of the mRNA expression levels of different genes in the injured kidneys compared with control kidneys is represented using volcano graphs. *P*<0.05 is considered statistically significant. Dotted lines represent *P*=0.05. (**C**) Representative images of renal sections stained with PAS or for RIPK3 and MLKL; Original magnification: ×100.

We further studied the changes in the expression of RN-related molecules in transient tissue injury. Since human and mice kidneys expressed relatively low mRNA levels of these molecules as in respective spleen, we selected three independent and well-characterized mouse models of acute kidney injury (AKI) viz. ischaemia-reperfusion injury (IRI), acute oxalate nephropathy and cisplatin nephropathy [11,32,34,35]. Tubular cell necrosis and inflammation are the hallmarks of kidney injury in these models (Supplementary Figure S1). We observed a consistent robust induction in the mRNA levels of necroptosis-related molecules viz. RIPK3, MLKL and CIAP1/2 in all three mouse models of AKI compared with the baseline (Figures 3A and 3B). In addition, CASP1, NLRP3 mRNA levels were significantly increased only in IRI and TNFR1, CASP8 mRNA levels were significantly increased only in acute oxalate nephropathy, whereas RIPK1, CASP8, FADD, CYPD and PARP1 mRNA levels were significantly decreased in cisplatin nephropathy (Figures 3A and 3B). GPX4 mRNA was not regulated in any of the three models (Figures 3A and 3B). Immunohistostaining of the kidneys for RIPK3 and MLKL confirmed the results at protein levels (Figure 3C). Together, acute tissue injuries involve consistent induction of molecules involved only in the necroptosis pathway, whereas the involvement of other RN pathways is highly dependent on the trigger.

### Regulated necrosis-related molecule expression in progressive tissue injury and fibrosis in mice

**Figure 4:**
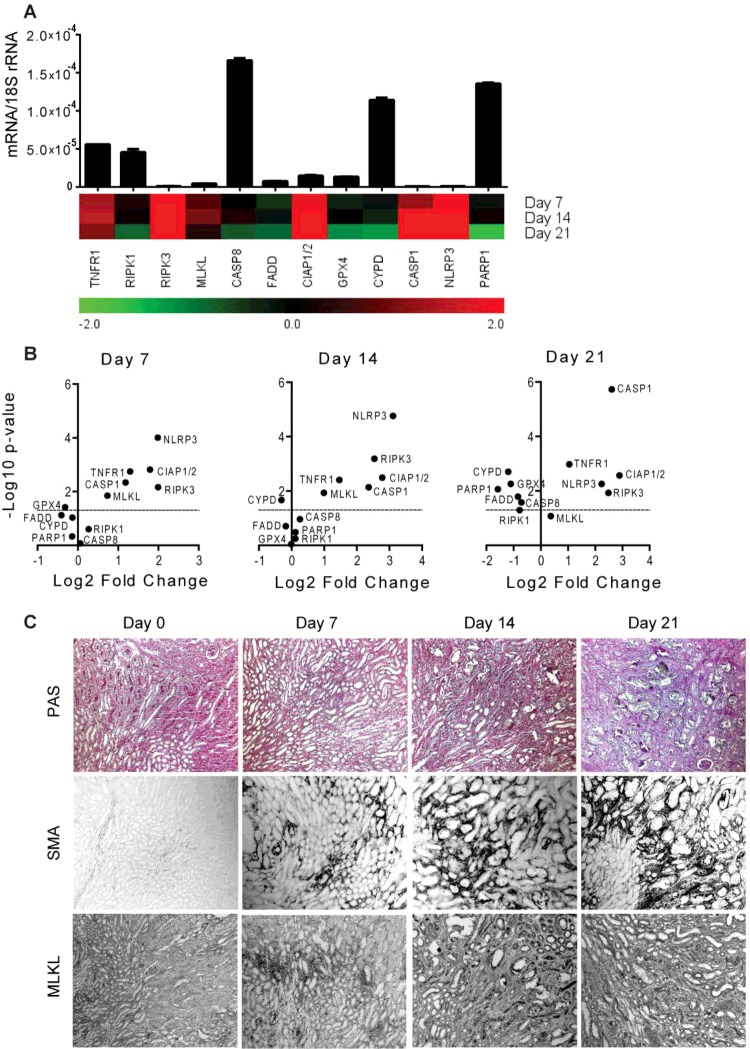
RN-related molecules mRNA expression in kidney remodelling in murine chronic oxalate nephropathy Chronic oxalate nephropathy was induced in mice as described in ‘Materials and Methods’ section. (**A**) Quantitative RT-PCR was performed on cDNAs derived from the kidneys of mice on day 7, 14 and 21. The mRNA expression levels were normalized to 18s rRNA expression level. The histogram represents the mRNA expression levels of different genes of the wild-type kidney (day 0). Data represent means ± S.E.M. The heatmap represents the relative expression of mRNA levels at day 7, 14 and 21 compared with day 0. (**B**) The statistical analysis of the mRNA expression levels of different genes in the kidneys at day 7, 14 and 21 compared with day 0 is represented using volcano graphs. *P*<0.05 is considered statistically significant. Dotted lines represent *P*=0.05. (**C**) Representative images of renal sections stained with PAS or for SMA and MLKL; Original magnification: ×100.

**Figure 5:**
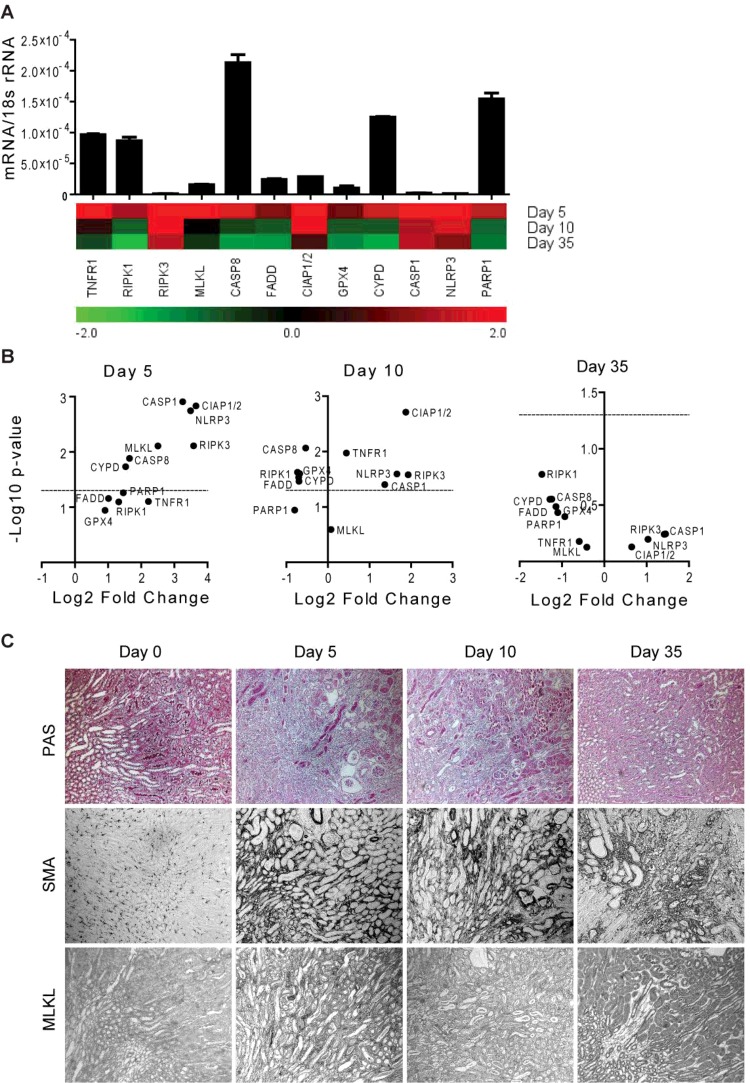
RN-related molecules mRNA expression in kidney remodelling in murine CKD after IRI CKD after unilateral renal pedicle clamping was induced in mice as described in ‘Materials and Methods’ section. (**A**) Quantitative RT-PCR was performed on cDNAs derived from the kidneys of mice on day 5, 10 and 35. The mRNA expression levels were normalized to 18s rRNA expression level. The histogram represents the mRNA expression levels of different genes of the contralateral kidney at day 5 (control). Data represent means ± S.E.M. The heatmap represents the relative expression of mRNA levels at day 5, 10 and 35 compared with control. (**B**) The statistical analysis of the mRNA expression levels of different genes in the kidneys at day 5, 10 and 35 compared with control is represented using volcano graphs. *P*<0.05 is considered statistically significant. Dotted lines represent *P*=0.05. (**C**) Representative images of renal sections stained with PAS or for SMA and MLKL; Original magnification: ×100.

Persistent and progressive tissue injury leads to the chronic tissue remodelling [41,42]. During this process, several inflammatory cells are recruited at the site of injury to maintain the inflammatory condition and promote tissue fibrosis by secreting pro-fibrotic mediators [43,44]. To study the induction of RN-related molecules in chronic tissue remodelling, we used two well-characterized murine models of chronic kidney disease (CKD) viz. progressive CKD model of chronic oxalate nephropathy [36] and CKD long after AKI model [45]. The mRNA levels of necroptosis- and pyroptosis-related molecules (TNFR1, RIPK3, MLKL, CIAP1/2, NLRP3 and CASP1) were significantly induced during the progression of chronic oxalate nephropathy, except for MLKL, which was decreased in the later phase (Figures 4A and 4B). RIPK1, CASP8, FADD, GPX4, CYPD and PARP1 mRNA expressions were significantly decreased in the later phase of chronic oxalate nephropathy (Figures 4A and 4B). In contrast with the progressive CKD model, we observed a very robust induction of all the RN-related molecule mRNA expression in the early phase of the CKD after AKI. The mRNA levels of RIPK3, NLRP3 and CIAP1/2 were as high as 11–12-fold compared with the baseline. The expression levels of all of these molecules declined with the progression of CKD after AKI (Figures 5A and 5B). The mRNA levels of necroptosis- and pyroptosis-related molecules (RIPK3, CIAP1/2, NLRP3 and CASP1) remained high, however statistically insignificant, even after 5 weeks (Figures 5A and 5B). Immunohistostaining of the kidneys for RIPK3 and MLKL confirmed the results at the protein level in progressive tissue fibrosis evident from smooth muscle actin (SMA) and collagen-1α1 (Figures 4C and 5C and Supplementary Figures S2 and S3). Together, the degrees of induction as well as the duration of the decline of the levels of RN-related molecules highly depend on the pathogenesis of the chronic tissue remodelling.

### Regulated necrosis-related molecule expression in systemic autoimmunity of MRL/lpr mice

**Figure 6:**
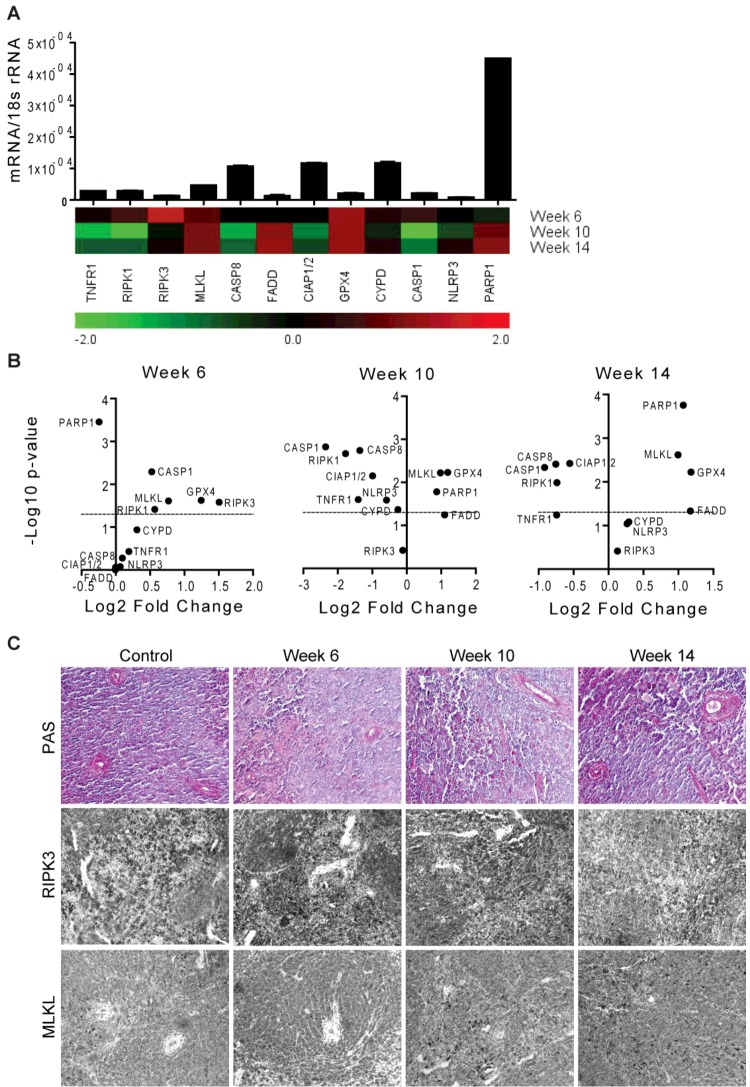
RN-related molecules mRNA expression in spleens of MRL/lpr mice MRL/lpr mice were used as a model of systemic autoimmunity. (**A**) Quantitative RT-PCR was performed on cDNAs derived from the spleens of mice on week 6, 10 and 14. The mRNA expression levels were normalized to 18s rRNA expression level. The histogram represents the mRNA expression levels of different genes of the MRL wild-type spleen (control). Data represent means ± S.E.M. The heatmap represents the relative expression of mRNA levels at week 6, 10 and 14 compared with control. (**B**) The statistical analysis of the mRNA expression levels of different genes in the spleens at week 6, 10 and 14 compared with control is represented using volcano graphs. *P*<0.05 is considered statistically significant. Dotted lines represent *P*=0.05. (**C**) Representative images of spleen sections stained with PAS or for RIPK3 and MLKL; Original magnification: ×200.

Systemic autoimmunity is characterized by high levels of circulating autoantibodies, tissue inflammation and progressive tissue remodelling of multiple organ systems [46–48]. To study the induction of RN-related molecules in systemic autoimmunity, we used the model of *Fas*-deficient MRL/lpr mice that displays spontaneous and similar to human systemic lupus erythematosus (SLE)-like systemic autoimmunity [37,46,49]. We observed that all of these molecules were constitutively expressed in the spleen of MRL-wild-type mice (Figure 6A). Furthermore, MLKL and GPX4 were expressed significantly high in the spleen during the disease progression. PARP1 mRNA was expressed significantly less at week 6, but its expression was increased significantly at week 10 and 14 (Figures 6A and 6B). FADD mRNA followed the similar trend of PARP1 and RIPK3 mRNA expression was significantly more at week 6 that was almost at the baseline at week 10 and 14 in the spleen (Figures 6A and 6B). Immunostaining of the spleen for RIPK3 and MLKL confirmed the results at the protein levels (Figure 6C). Thus, mostly the expression of RN-related molecules is down-regulated except for MLKL, CYPD, PARP1 and FADD in the spleen during the progression of SLE.

### Regulated necrosis-related molecule expression in progressive lupus nephritis of MRL/lpr mice

**Figure 7:**
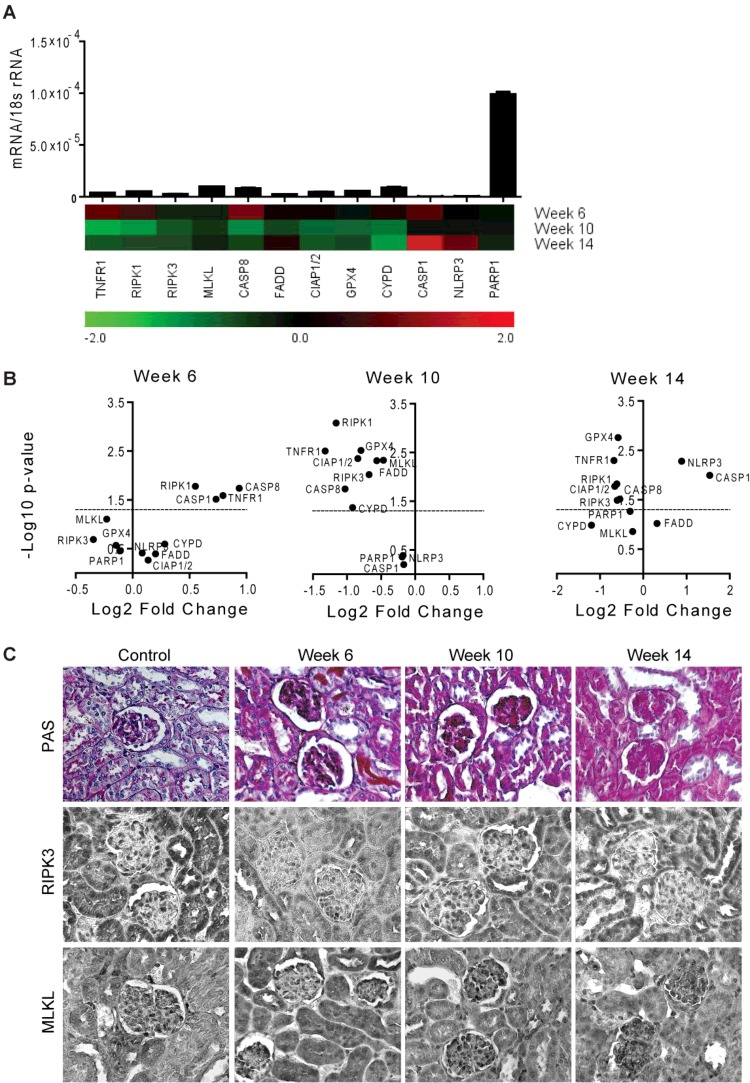
RN-related molecules mRNA expression in kidneys of MRL/lpr mice (**A**) Quantitative RT-PCR was performed on cDNAs derived from the kidneys of mice on week 6, 10 and 14. The mRNA expression levels were normalized to 18s rRNA expression level. The histogram represents the mRNA expression levels of different genes of the MRL wild-type kidneys (control). Data represent means ± S.E.M. The heatmap represents the relative expression of mRNA levels at week 6, 10 and 14 compared with control. (**B**) The statistical analysis of the mRNA expression levels of different genes in the kidneys at week 6, 10 and 14 compared with control is represented using volcano graphs. *P*<0.05 is considered statistically significant. Dotted lines represent *P*=0.05. (**C**) Representative images of renal sections stained with PAS or for RIPK3 and MLKL; Original magnification: ×400.

The most frequent complication of SLE is an immune complex glomerulonephritis called lupus nephritis (LN). The pathogenesis of progressive renal remodelling in LN involves a variety of extra- and intra-renal pathomechanisms [50]. Therefore, we also assessed the expression of RN-related molecules in the kidneys during the progression of LN. We observed that all of these molecules were constitutively expressed in the kidney of MRL-wild-type mice (Figure 7A). Further, all RN-related molecule mRNA levels were down-regulated with the progression of renal remodelling in LN except for the molecules involved in pyroptosis e.g. CASP1 and NLRP3, which were significantly high at week 14 and FADD that was moderately increased (Figures 7A and 7B). Unlike spleen, the mRNA expression of MLKL and PARP1 remained unchanged and GPX4 was significantly down-regulated at all-time points (Figures 7A and 7B). The expressions of TNFR1, RIPK1 and CASP8 were significantly up-regulated at week 6 and were down-regulated significantly at week 10 and 14 along with RIPK3 and CIAP1/2 (Figures 7A and 7B). We also checked the protein expression of some of these genes in the kidneys. Immunostaining of the kidney for RIPK3 and MLKL confirmed the results at the protein levels (Figure 7C). Thus, similar to spleen the expression of RN-related molecules is down-regulated except for CASP1 and NLRP3 in the kidney during the progression of tissue remodelling in LN.

## DISCUSSION

Historically, necrosis was viewed as an uncontrolled form of cell death however, more recent studies showed that necrosis can be a highly regulated process [2,3]. Based, on the pathways involved, RN was classified into different categories [2,3]. Tissue injury and inflammation are tightly associated processes, for example, necrosis is associated with the release of intracellular danger associated molecules (DAMPs), which induce inflammatory cytokine release from the immune cells by activating TLRs [51]. Further, certain cytokines also possess the capacity to induce RN [12]. This auto-amplification loop between RN and inflammation is also referred as necroinflammation [23,24]. Our data demonstrate that the relative mRNA expression profiles of the RN-related molecules differ a lot between humans and mice as it has been already described for the PRRs [28–31].

Necroinflammation is the central pathomechanism of ischaemic and toxic AKI [11,24,25,32,34,35,52]. The induction of RIPK3, MLKL and CIAP1/2 in murine models of renal IRI, acute oxalate nephropathy and cisplatin nephropathy suggest the involvement of necroptosis as the main mechanism of acute tubular necrosis (ATN) despite different triggers of injury–for example, hypoxia, calcium oxalate crystals and a toxic chemical respectively. Although the disease pathology is similar in these models, the expression of other RN-related molecules, e.g. TNFR1, RIPK1, CASP8, FADD, CYPD, CASP1, NLRP3 and PARP1 etc. are different. This suggests that different pathomechanisms may lead to alike disease pathology. Interestingly, although CYPD was not induced in cisplatin nephropathy as well as CASP1, NLRP3 were not induced in acute oxalate nephropathy, *CypD*-, *Nlrp3-* and *Casp1-*deficient mice show less ATN than wild-type mice [19,34]. Furthermore, although GPX4 mRNA levels were not much regulated, GPX4 protein depletion-related ferroptosis has been confirmed as a mode of tubular cell death in IRI and acute oxalate nephropathy [53,54]. Therefore, it is obvious that mRNA profiles do not always predict the functional contribution of these molecules in disease processes. Nevertheless, we observed a robust induction of RIPK3 and MLKL, two core proteins of the necroptosis pathway, which was already shown to contribute to ATN in all three models of AKI [11,19,35], hence the functional contribution of other induced genes should be tested.

Unlike acute tissue injuries, the contribution of necroinflammation in the chronic tissue injury is not so clear. The presence of leucocytes rather represents tissue repair or fibrosis [55,56]. The chronic disease processes are characterized by low grade of persistent inflammation and a more chronic wound healing kind of tissue response involving tissue atrophy and scaring. We studied progressive tissue fibrosis using a murine model of chronic oxalate nephropathy, as well as CKD after IRI model. Although the kinetics of up-regulation, as well as subsequent down-regulation, was specific to disease pathology, both models showed induction of RN-related molecules in the early phase of the disease. This means that the maximum protective effect of cell death inhibitors might only be achieved if applied in the early phase of progressive tissue fibrosis. In addition, our data suggest that inhibitors of necroptosis, ferroptosis or pyroptosis may also be beneficial in delaying the progression of fibrosis, if applied in the later phase, especially after acute injury. Furthermore, it will be interesting to study the impact of these molecules on the macrophage phenotype switch during progressive fibrosis.

SLE is characterized persistent exposure to autoantigens as a result of alterations in cell death pathways e.g. apoptosis, NETosis etc., and clearance deficiency of dead cells [47,48]. The loss of self-tolerance and production of autoantibodies by autoreactive lymphocytes is the hallmark of SLE [47,48]. This is why we mostly did not observe any regulation of RN-related molecules in kidneys of MRL/lpr mice, except for CASP1 and NLRP3. However, the induction of these molecules is rather protective as they have been demonstrated to suppress SLE and LN [57]. Our use of *Fas*-deficient MRL/lpr mice as a model of SLE may explain the up-regulation of FADD mRNA in the spleen. We also observed an up-regulation in the mRNA expression of MLKL, GPX4 and PARP1 in the spleen. Interestingly, the peripheral blood mononuclear cells of patients with SLE as well as systemic sclerosis are known to express low levels and activity of PARP1 [58–60]. However, the specific contribution of these molecules in SLE has not yet been studied in detail.

The present study did not focus on the gender-specific differences within the same species. However, several studies have addressed the possible role of gender as a biological variable–for example, lung and kidney injury, gut microbiota composition, haematopoiesis etc. [61–65]. Therefore, the gender-specific differences, along with organ- and species-specific differences, also deserve a careful consideration.

Together, we have identified significant differences in the mRNA expression profiles of the RN-related molecules in humans and murine solid organs, as well as their regulation in murine autoimmunity, acute tissue injury and progressive tissue fibrosis. Therefore, it is important to take into account these species-specific differences to avoid misinterpretation and wrong conclusions. These findings warrant the need to validate the functional roles of these molecules, as identified in rodent studies, in human studies as well.

## Abbreviations

AKI: acute kidney injury
ATN: acute tubular necrosis
CIAP: cellular inhibitor of apoptosis protein
CASP: caspase
CKD: chronic kidney disease
CYPD: cyclophilin D
dNTP: deoxyribonucleotide
FADD: Fas-associated protein with death domain
GAPDH: glyceraldehyde-3-phosphate dehydrogenase
IRI: ischaemia-reperfusion injury
LN: lupus nephritis
MLKL: mixed lineage kinase domain-like
MPT: mitochondria permeability transition
NET: neutrophil extracellular trap
NLRP3: nod-like receptor family pyrin domain containing 3
PARP1: poly(ADP-ribose) polymerase-1
PAS: periodic acid–Schiff
PRR: pattern recognition receptor
qRT-PCR: quantitavie real time polymerase chain reaction
RHIM: RIP homotypic interaction motif
RIPK: receptor activated protein kinase
RN: regulated necrosis
SLE: systemic lupus erythematosus
SMA: smooth muscle actin
TLR: toll-like receptor
TNFR1: tumour necrosis factor receptor-1
